# Dietary proanthocyanidins boost hepatic NAD^+^ metabolism and SIRT1 expression and activity in a dose-dependent manner in healthy rats

**DOI:** 10.1038/srep24977

**Published:** 2016-04-22

**Authors:** Gerard Aragonès, Manuel Suárez, Andrea Ardid-Ruiz, Maria Vinaixa, Miguel A. Rodríguez, Xavier Correig, Lluís Arola, Cinta Bladé

**Affiliations:** 1 Nutrigenomics Research Group, Department of Biochemistry and Biotechnology, Universitat Rovira i Virgili, Tarragona, Spain; 2 Metabolomics Platform of the Spanish Biomedical Research Center in Diabetes and Associated Metabolic Disorders (CIBERDEM), University Rovira i Virgili, IISPV, Reus, Spain; 3 Center for Omic Sciences (COS), Universitat Rovira i Virgili, Reus, Spain

## Abstract

Proanthocyanidins (PACs) have been reported to modulate multiple targets by simultaneously controlling many pivotal metabolic pathways in the liver. However, the precise mechanism of PAC action on the regulation of the genes that control hepatic metabolism remains to be clarified. Accordingly, we used a metabolomic approach combining both nuclear magnetic resonance and mass spectrometry analysis to evaluate the changes induced by different doses of grape-seed PACs in the liver of healthy rats. Here, we report that PACs significantly increased the hepatic nicotinamide adenine dinucleotide (NAD^+^) content in a dose-dependent manner by specifically modulating the hepatic concentrations of the major NAD^+^ precursors as well as the mRNA levels of the genes that encode the enzymes involved in the cellular metabolism of NAD^+^. Notably, *Sirtuin 1* (*Sirt1*) gene expression was also significantly up-regulated in a dose-response pattern. The increase in both the NAD^+^ availability and *Sirt1* mRNA levels, in turn, resulted in the hepatic activation of SIRT1, which was significantly associated with improved protection against hepatic triglyceride accumulation. Our data clearly indicates that PAC consumption could be a valid tool to enhance hepatic SIRT1 activity through the modulation of NAD^+^ levels.

Natural dietary polyphenols and specifically proanthocyanidins (PACs), the most structurally complex subclass of flavonoids, are bioactive food compounds that are primarily present in fruits and vegetables and exhibit many protective effects against cardiovascular disease^1^. In this context, our group and others have reported many healthy and beneficial effects of PACs on different metabolic syndrome-related pathologies, such as insulin resistance, dyslipidemia, obesity, hypertension and inflammation^2^,^3^,^4^,^5^,^6^,^7^. Furthermore, other studies performed at the molecular level have demonstrated that PACs could play an important role in the regulation of the transcriptional networks that control various critical metabolic processes in the liver. Specifically, PAC consumption was shown to protect the liver from lipid accumulation by reducing the expression of target lipogenic genes and up-regulating fatty acid oxidation^8^,^9^,^10^.

Several mechanisms by which PACs reduce these hepatic metabolic disturbances have been described, such as the direct interaction with intracellular signaling pathways^11^ and modulation of epigenetic factors, including both microRNAs^12^,^13^ and components of the DNA methylation machinery^14^. However, the actual molecular mechanisms involved in the health benefits of PAC consumption in the liver remain mostly speculative and the global mechanism of action is still largely unknown. This might be because previous studies have primarily focused on genomic or proteomic changes instead of assessing the direct changes in hepatic metabolites. Certainly, metabolomics, one of the most rapidly growing fields of contemporary science^15^, might be a good alternative to characterize the hepatic metabolites that are modified as a result of exogenous challenges, such as PAC consumption, and provide new evidence linking the cellular pathways to the biological mechanisms.

In this context, two different technologies have the potential to discover the metabolic alterations in liver samples: nuclear magnetic resonance (NMR) and mass spectrometry (MS)^16^,^17^,^18^. However, to the best of our knowledge, there are no previous metabolomic studies in the literature aimed at evaluating the effect of PAC consumption on liver metabolites. Therefore, here, we performed a multiplatform approach combining both NMR and MS metabolomic analysis of the liver of healthy rats that were chronically supplemented with different doses of PACs. Because homeostasis is very robust, challenging homeostasis is more informative than static homeostatic studies^19^. Therefore, we aimed to quantitatively identify the alterations in hepatic metabolites in response to a fat overload challenge to further elucidate the mechanism by which PAC consumption can modulate lipid metabolism in liver. Our results revealed that PAC consumption could be relevant for improving hepatic lipid metabolism in an *in vivo* model by regulating the liver’s response through a metabolic increase in both cellular nicotinamide adenine dinucleotide (NAD^+^) availability and sirtuin activity.

## Results

### Metabolomics revealed that PAC consumption robustly increased the hepatic NAD^+^ levels in a dose-response manner

**Figure 1:**
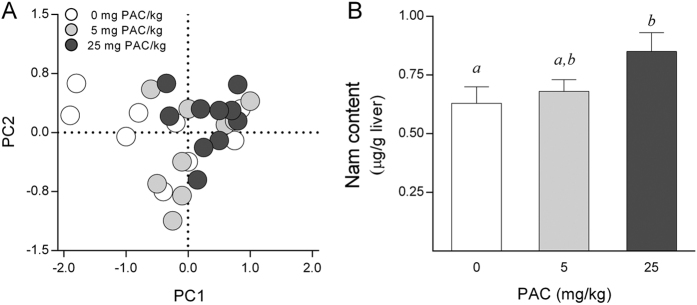
Metabolic profile assessed by untargeted H^1^NMR in the liver of healthy rats supplemented with different doses of PACs. (**A**) The PC analysis did not show statistically significant changes in liver metabolic profile following PAC consumption. (**B**) The animals supplemented with 25 mg of PACs/kg bw displayed higher hepatic Nam levels compared to the control group. The rats were fed a standard chow diet supplemented with 0 (control group), 5 or 25 mg of PACs/kg of body weight for 21 days. The values shown are the means ± SE of 10 animals per group. The letters denote a significant difference between groups (*P* < 0.05; one-way ANOVA and Tukey’s HSD post-hoc test comparison). Nam: nicotinamide; PACs: proanthocyanidins; PC: principal component.

The liver metabolic profile changes associated with PAC consumption were initially assessed only in the liver samples from animals receiving 0, 5 and 25 mg of PAC/kg body weight (bw) using an untargeted ^1^H-NMR-based metabolomics approach. A total of 46 spectral regions were identified and quantified from the ^1^H-NMR spectra acquired from liver extracts. Then, a multivariate principal components analysis (PCA) was performed on the resulting data for exploratory purposes. The PCA score plot accounted for a 57% variance of the original matrix and did not show any clear clustering trend of the data according to the PAC consumption groups (Fig. 1A). Subsequently, we used a one variable at a time 1-way ANOVA to compare the PAC consumption groups. The concentrations of fumaric acid (singlet, 2 × CH, δ = 6.5 ppm) were slightly decreased as a consequence of PAC consumption (*P* = 0.04 by Tukey’s HSD post-hoc test), whereas the hepatic nicotinamide (Nam) levels (singlet, H-2, δ = 8.9 ppm & doublet, H-6, δ = 8.7 ppm) were robustly increased in those animals supplemented with 25 mg of PAC/kg bw (Fig. 1B).

**Figure 2:**
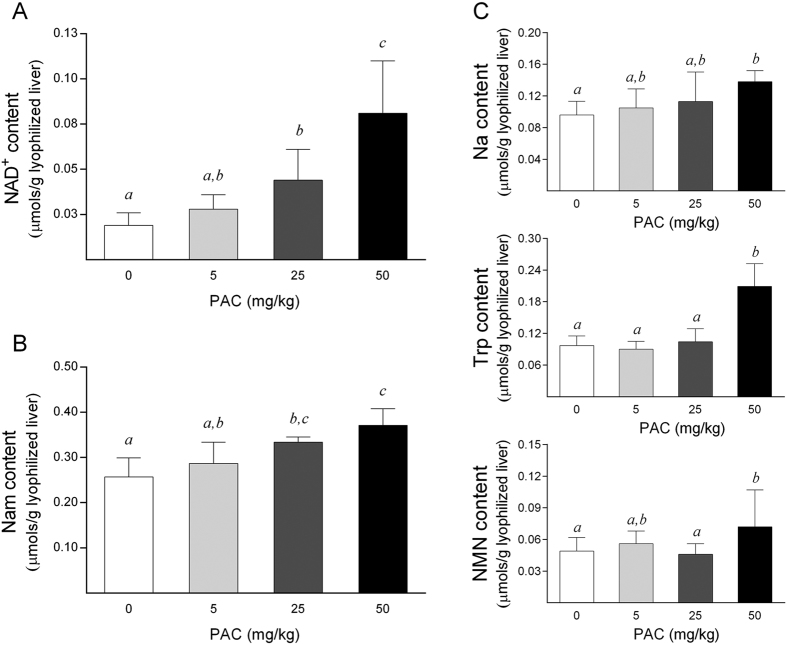
Hepatic levels of NAD^+^ and the main NAD^+^ precursors in healthy rats supplemented with different doses of PACs. (**A–B**) PAC consumption increased the hepatic NAD^+^ (**A**) and Nam (**B**) levels in a dose-dependent manner. (**C**) Similarly, the Trp, Na and NMN concentrations were also significantly increased in animals supplemented with 50 mg of PACs/kg bw compared to the control rats. The animals were fed a standard chow diet supplemented with 0 (control group), 5, 25 or 50 mg of PACs/kg bw for 21 days. The values shown are the means ± SE of 10 animals per group. The letters denote a significant difference between groups (*P* < 0.05; one-way ANOVA and LSD post-hoc test comparison). Na: nicotinic acid; NAD^+^: nicotinamide adenosine dinucleotide; Nam: nicotinamide; NMN: nicotinamide mononucleotide; PACs: proanthocyanidins; Trp: tryptophan.

Next, as Nam is one of the main liver precursors of NAD^+^, these results were validated and complemented by quantifying the NAD^+^ levels with targeted analyses of the liver samples using triple-quadrupole mass spectrometry (QqQ-MS/MS). Notably, in order to further investigate the linear dose-dependent related effects of PAC consumption, additional liver samples from another group of animals receiving a higher dose of PAC (50 mg/kg bw) were also evaluated in targeted analyses. Accordingly, we confirmed that PAC consumption significantly increased the hepatic NAD^+^ levels in a dose-dependent manner (Fig. 2A), indicating that PAC administration could be a valid tool to boost the cellular NAD^+^ content in the liver. In addition, the hepatic Nam levels were clearly higher in the PAC-supplemented animals compared to the control group (Fig. 2B), as previously observed by global NMR. Then, we tested whether the increase in the NAD^+^ and Nam content would be paralleled by changes in the other major liver metabolites involved in the NAD^+^ cycle. Accordingly, the tryptophan (Trp), nicotinic acid (Na) and nicotinamide mononucleotide (NMN) concentrations were also significantly increased in animals supplemented with 50 mg of PAC/kg bw compared to the control rats (Fig. 2C). Additionally, a positive and significant relationship between the hepatic NAD^+^ concentrations and the levels of their major immediate precursors in the liver, Trp (*rho* = 0.622, *P* < 0.001) and Nam (*rho* = 0.604, *P* < 0.001), was also observed, suggesting that the increase in the hepatic NAD^+^ content is due to changes of these two NAD^+^ precursors. Finally, other liver intermediates and the end-products of NAD^+^ catabolism were also measured. However, the nicotinamide riboside (Nr), nicotinic acid mononucleotide (NaMN), nicotinic acid adenine dinucleotide (NaAD), nicotinamide *n*-oxide, nicotinuric acid and *n*-methylnicotinamide levels did not display significant differences among the groups (data not shown) or were below the limit of detection in all groups of animals.

### PAC consumption induced an overexpression of the genes that encode the enzymes involved in the *de novo* NAD^+^ biosynthesis pathway

**Figure 3:**
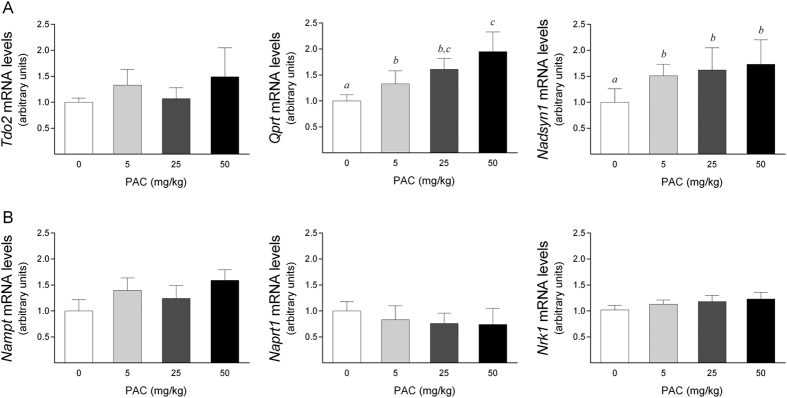
Hepatic mRNA levels of selected genes of NAD^+^ biosynthesis in healthy rats supplemented with different doses of PACs. (**A,B**) PAC consumption up-regulated the mRNA levels of specific genes involved in de novo (**A**), but not the salvage (**B**) NAD^+^ biosynthetic pathways. The mRNA levels of the selected genes were normalized to the Ppia mRNA levels. The animals were with a standard chow diet supplemented with 0 (control group), 5, 25 or 50 mg of PACs/kg bw for 21 days. The values shown are the means ± SE of 10 animals per group. The letters denote a significant difference between groups (*P* < 0.05; one-way ANOVA). NAD^+^: nicotinamide adenosine dinucleotide; Nadsyn1: NAD^+^ synthetase; Nampt: nicotinamide phosphoribosyltransferase; Naprt1: nicotinate phosphoribosyltransferase 1; Nrk1: nicotinamide riboside kinase1; PACs: proanthocyanidins; Qprt: quinolinate phosphoribosyltransferase; Tdo2: tryptophan 2,3-dioxygenase.

To analyze these changes further, we next evaluated whether the enhanced NAD^+^ levels upon PAC consumption could be derived from increased NAD^+^ biosynthesis. Thus, we determined the mRNA levels of the major enzymes of the NAD^+^ biosynthetic pathways. The first step of the *de novo* NAD^+^ biosynthesis pathway is the conversion of Trp into N-formylkynurenine through an enzymatic reaction catalyzed by tryptophan 2,3-dioxygenase (Tdo2). N-formylkynurenine is then directed to spontaneous cyclization to quinolinic acid, which is converted to NaMN through quinolinate phosphoribosyltransferase (Qprt) activity. NaMN is then transformed to NaAD by the nicotinamide mononucleotide adenylyltransferase (Nmnat) enzymes, and NaAD is finally amidated to NAD^+^ by NAD^+^ synthetase 1 (Nadsyn1). Thus, we examined the effect of PAC consumption on the levels of the *Tdo2, Qprt* and *Nadsyn1* mRNAs. Although we could not detect differences in the *Tdo2* mRNA levels between the animal groups, PAC consumption significantly up-regulated the *Qprt* and *Nadsyn1* mRNA levels in a dose-dependent manner (Fig. 3A). Notably, the increased levels of these transcripts were already significant at a dose of 5 mg of PAC/kg bw, indicating that this dose was sufficient to efficiently increase the mRNA levels of the major *de novo* NAD^+^ -biosynthetic enzymes.

Alternatively, NAD^+^ is also synthesized through the NAD^+^ *salvage* pathway from its precursors Na, Nam and Nr. Beginning with Na, the first step in NAD^+^ synthesis is catalyzed by nicotinic acid phosphoribosyltransferase 1 (Naprt1) and leads to the formation of NaMN. Similarly, Nam is converted to NMN by nicotinamide phosphoribosyltransferase (Nampt); NMN is also the product of phosphorylation of Nr by nicotinamide riboside kinase 1 (Nrk1). Both NaMN and NMN are then converted to NaAD by Nmnat, after which the NaMN-derived NaAD requires final amidation through Nadsyn1. Importantly, our results showed that PAC consumption did not modify the levels of the *Nampt, Naprt1* or *Nrk1* mRNAs (Fig. 3B). In fact, we could not even detect a statistically significant difference at a dose of 50 mg of PAC/kg bw compared to the control group, indicating that the higher NAD^+^ levels observed in the livers of the PAC-supplemented animals were not due to an increase in direct NAD^+^ synthesis from Nam, Na or Nr. Together, these results indicated that PAC consumption increases the cellular NAD^+^ content by a direct effect on *de novo* NAD^+^ biosynthesis rather than by indirectly affecting the major NAD^+^ *salvage* pathway.

### PAC consumption modified the activity of the major NAD^+^ -consuming enzymes by simultaneously down-regulating the hepatic levels of the *Parp1* and *Cd38* mRNAs

**Figure 4:**
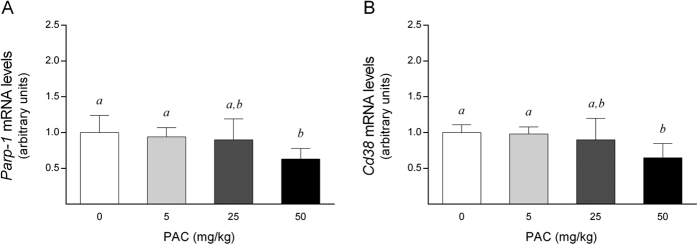
Hepatic mRNA levels of selected genes of NAD^+^ consumption in healthy rats supplemented with different doses of PACs. (**A,B**) PAC consumption down-regulated the mRNA levels of two major enzymes, *Parp1* (A) and *Cd38* (**B**), implicated in NAD^+^ consumption. The mRNA levels of the selected genes were normalized to the *Ppia* mRNA levels. The animals were fed a standard chow diet supplemented with 0 (control group), 5, 25 or 50 mg of PACs/kg bw for 21 days. The values shown are the means ± SE of 10 animals per group. The letters denote a significant difference between groups (*P* < 0.05; one-way ANOVA). Cd38: cluster of differentiation 38, also known as cyclic ADP ribose hydrolase; NAD^+^: nicotinamide adenosine dinucleotide; PACs: proanthocyanidins; Parp1: poly(ADP-ribose) polymerase 1.

As ADP-ribosylation reactions consume NAD^+^, we also analyzed whether the mRNA levels of the major genes involved in this process, *Poly(ADP-ribose) polymerase 1* (*Parp1*) and *Cyclic ADP-ribose hydrolases* (*Cd38*), could also contribute to the increase in the NAD^+^ levels. Notably, at dose of 50 mg/kg bw, PAC consumption significantly down-regulated the *Parp1* and *Cd38* mRNA levels compared to the control animals (*P = *0.04 and *P* = 0.014, respectively; Mann-Whitney test), without any effect at lower doses (Fig. 4). PARP1 and CD38 are the major NAD^+^ -consuming enzymes, thus the decrease in their activities may have also contributed to the increase in the hepatic NAD^+^ content. Indeed, there was a negative and significant correlation between the hepatic NAD^+^ concentrations and the *Parp1* mRNA levels (*rho* = −0.661, *P* = 0.004) that was not as evident for the *Cd38* mRNA values (*rho* = −0.457, *P* = 0.075).

### PAC consumption robustly enhanced both the hepatic *Sirt1* mRNA levels and SIRT1 activity in a dose-dependent manner

**Figure 5:**
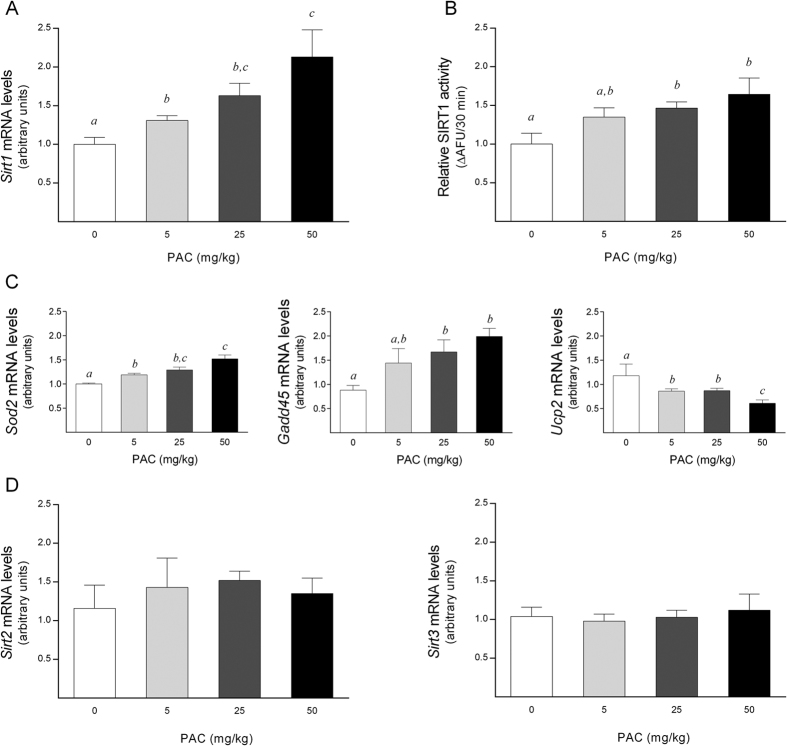
Hepatic SIRT activity in healthy rats supplemented with different doses of PACs. (**A,B**) PAC consumption dose-dependently increased the hepatic *Sirt1* mRNA levels (**A**) and SIRT1 activity (**B**). (**C**) The mRNA levels of *Gadd45* and *Sod2* were significantly up-regulated in a dose-dependent manner, whereas the *Ucp2* mRNA levels were down-regulated in a similar manner. (**D**) There was no statistically significant effect of PAC consumption on the hepatic *Sirt2* o*r Sirt3* mRNA levels. SIRT activity was indirectly assessed by determining both the mRNA levels of *Sirt1, Sirt2* and *Sirt3* and the mRNA levels of selected specific genes modulated by FOXO1 activity. The mRNA levels of the selected genes were normalized to the *Ppia* mRNA levels. The animals were fed a standard chow diet supplemented with 0 (control group), 5, 25 or 50 mg of PACs/kg bw for 21 days. The values shown are the means ± SE of 10 animals per group. The letters denote a significant difference between groups (*P* < 0.05; one-way ANOVA). AFU: arbitrary fluorescence units; Gadd45: growth arrest and DNA damage-inducible 45; PACs: proanthocyanidins; Sirt: sirtuin; Sod2: superoxide dismutase 2; Ucp2: uncoupling protein 2.

The ability of PAC to increase the intracellular NAD^+^ levels *in vivo* prompted us to test whether it could also modulate the sirtuin 1 (SIRT1) levels. PAC consumption resulted in a significant up-regulation of the *Sirt1* mRNA levels in a dose-dependent manner (Fig. 5A). Moreover, we observed a positive and significant relationship between the *Sirt1* mRNA levels and the hepatic NAD^+^ content (*rho* = 0.493, *P* = 0.03). The combination of higher NAD^+^ concentrations with higher *Sirt1* mRNA levels provides an excellent *scenario* for increased SIRT1 activity. Accordingly, SIRT1 activity was increased in a dose-response manner, with a significant activation in the liver of rats treated with 25 or 50 mg PACs/kg bw (Fig. 5B).

Moreover, indirect measurements of SIRT1 activity also reinforced the actual activation of SIRT1 in liver by PACs. Considering that the deacetylation of forkhead O-box protein 1 (FOXO1) is mediated by SIRT1, we evaluated the transcriptional activation of FOXO1 by determining the mRNA levels of its target genes *Superoxide dismutase 2* (*Sod2*) and *Growth Arrest and DNA Damage* (*Gadd45*) to confirm this hypothesis^20^. Accordingly, their mRNA levels were significantly up-regulated in the livers of the PAC-supplemented animals in a dose-dependent manner (Fig. 5C). Consistent with SIRT1 being a negative regulator of *Mitochondrial uncoupling protein 2* (*Ucp2*) expression^21^, PAC consumption significantly decreased the *Ucp2* mRNA levels in a similar dose-dependent pattern (Fig. 5C). Finally, as the altered NAD^+^ levels could also potentially impact other SIRT proteins, we also tested the levels of *Sirt2* and *Sirt3* transcripts, which act as cytosolic and mitochondrial sirtuins, respectively. However, no changes were observed in the mRNA levels of these genes with respect to control group (Fig. 5D).

### PAC consumption improved the hepatic lipid content in both healthy rats and cultured cells in a SIRT1-mediated manner

**Figure 6:**
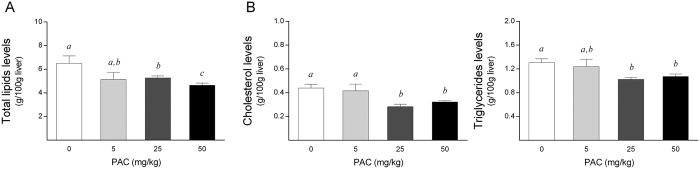
Hepatic lipid profile in healthy rats supplemented with different doses of PACs. (**A,B**) PAC consumption decreased the total lipid accumulation in the liver (**A**) by accordingly diminishing the hepatic cholesterol and triglyceride concentrations (**B**). The animals were fed a standard chow diet supplemented with 0 (control group), 5, 25 or 50 mg of PACs/kg bw for 21 days. The values shown are the means ± SE of 10 animals per group. The letters denote a significant difference between groups (*P* < 0.05; one-way ANOVA). PACs: proanthocyanidins.

Given the promising role of SIRT1 in protecting against metabolic liver diseases, we next evaluated the effects of PAC consumption on the liver fat content in animals subjected to an acute dietary fat challenge. Confirming our hypotheses, PACs significantly reduced the hepatic lipid accumulation in a dose-dependent manner (Fig. 6A), and this was associated with a significant decrease in both the hepatic cholesterol and triglyceride concentrations (Fig. 6B). Notably, the hepatic lipid concentrations were negatively and significantly related to the *Sirt1* mRNA levels in the PAC-supplemented animals (*rho* = −0.678, *P* = 0.002). Moreover, when we applied a multivariate linear regression model to assess the role of the variables known to influence the hepatic lipid concentrations, the *Sirt1* mRNA levels were the only variable that was significantly associated with fat accumulation in the liver, with a β coefficient of −0.896 (*P* = 0.003), highlighting the major role of SIRT1 in reducing the hepatic fat depots in the PAC-supplemented animals.

**Figure 7:**
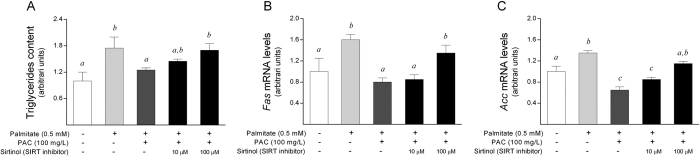
Effect of PACs on an experimental model of hepatocellular steatosis in HepG2 cells. (**A**) The PAC-treated cells exhibited decreased triglyceride levels. (**B,C**) The mRNA levels of lipogenic enzymes were assessed by RT-qPCR, and the *Fas* (**B**) and *Acc* (**C**) mRNA levels were significantly down-regulated in the treated cells compared to the untreated cells. (**A,B**) When the cultured cells were treated with sirtinol, the protective effect of PACs on our experimental model was significantly attenuated. The HepG2 cells were incubated with a mixture of palmitate 0.5 mM and glucose 30 mM for 48 hours to induce fat-overloading. Then, the cells were treated with 100 mg/L PACs for the last 24 hours of incubation in the presence or absence of sirtinol, a SIRT1 inhibitor. All values represent the means of three independent experiments. The letters denote a significant difference between groups (*P* < 0.05; one-way ANOVA). Acc: acetyl-coA carboxylase; Fas: fatty acid synthase; PACs: proanthocyanidins.

To further solidify our data, we also evaluated the efficacy of PACs in protecting against fat overaccumulation in an experimental *in vitro* model. Thus, HepG2 cells were incubated with a mixture of 0.5 mM palmitate and 30 mM glucose for 48 hours to induce fat-overloading. Then, the cells were treated with 100 mg/L PACs for the last 24 hours of incubation. Indeed, when the cultured cells were treated with PACs, the intracellular concentrations of triglycerides decreased significantly to basal levels, reversing the fat accumulation observed in the untreated cells (Fig. 7A). In addition, to confirm the specificity of SIRT1 in attenuating the triglyceride accumulation in cultured cells, the HepG2 cells were also treated with sirtinol, a SIRT1 inhibitor, for 24 hours. Notably, the protective effect of PACs on cellular triglyceride accumulation was significantly abrogated when the cells were treated with 100 μM sirtinol, and the triglyceride levels did not significantly decrease after the PAC treatment (Fig. 7A). Finally, when the mRNA levels of the key genes involved in lipid metabolism were assessed by real time quantitative polymerase chain reaction (RT-qPCR), PAC treatment resulted in decreased *Fatty acid synthase* (*Fas*) and *Acetyl-coA carboxylase* (*Acc*) mRNA levels compared to the untreated cells, indicating that the delipidating effect of the PAC treatment may be mediated by limiting the capacity for *de novo* lipogenesis (Fig. 7B,C). In addition, when the cultured cells were treated with 100 μM sirtinol, the levels of the *Fas* and *Acc* mRNAs were again not affected by the PAC treatment, thus confirming that SIRT1 activation plays a critical role in regulating hepatic *de novo* lipogenesis and, consequently, fat accumulation in this experimental model.

## Discussion

Several molecular mechanisms have been described to account for the metabolic benefits of PAC consumption^8^,^10^,^11^,^12^,^13^. However, all of these mechanisms only partially account for all the physiological and biochemical effects of PACs. Therefore, in this study, we have used a combination of metabolomics and gene expression analyses to identify the potential global mechanism that could explain the diverse metabolic effects of PAC consumption. Interestingly, comparative liver metabolomics revealed that NAD^+^ and its metabolites are clear targets of PAC consumption. Certainly, PAC administration dose-dependently increased both the hepatic NAD^+^ content and mRNA levels of the genes involved in the NAD^+^ biosynthesis pathway, highlighting NAD^+^ homeostasis as potential PACs target in the livers of healthy rats.

Notably, other studies of our group indicate that an acute single dose of PACs is also able to modulate hepatic NAD^+^ levels^22^. Thus, altogether, these results reveal NAD^+^ homeostasis as a major target of PACs in liver.

NAD^+^ plays a pivotal role in cells, as it is indispensable for energy production and acts as reusable coenzyme in metabolic redox reactions. The roles of NAD^+^, however, have expanded beyond its role as a coenzyme, as NAD^+^ and its metabolites also act as degradation substrates for a wide range of enzymatic reactions that catalyze ADP-ribosylation and deacetylation of proteins^23^. Through these activities, NAD^+^ links the cellular metabolism to changes in signaling and transcriptional events. In addition, different studies have unequivocally demonstrated the ability of NAD^+^ to dynamically respond to physiological stimuli, such as exercise^24^ and caloric restriction^25^. Thus, the modulation of NAD^+^ homeostasis by PAC consumption empowers these compounds to modify a large number of metabolic processes in the liver. Furthermore, the liver exports NAD^+^ precursors to other organs, such as the brain and muscle, which have a lower capacity to synthesize NAD^26^. Thus, as several organs depend on NAD^+^ homeostasis in the liver, the modulation of NAD^+^ homeostasis by PACs could be extended as a general effect in the whole organism.

**Figure 8:**
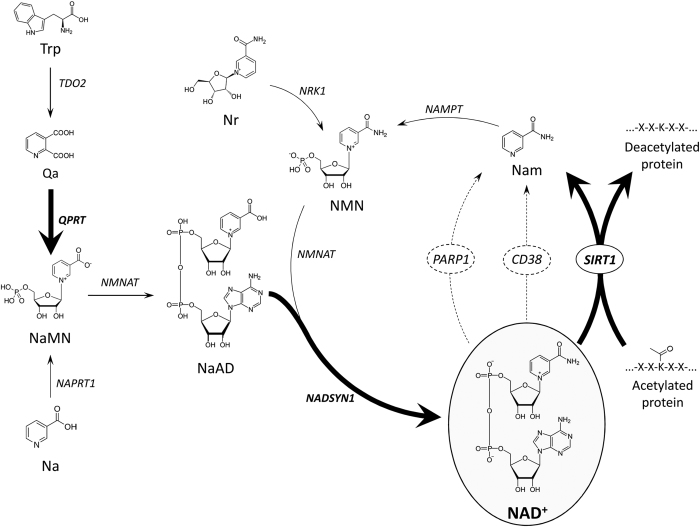
Schematic representation of mammalian NAD^+^ metabolism and its modulation by PACs. Bold lines: activated reaction; dashed lines: repressed reaction. Na: nicotinic acid; NAD^+^: nicotinamide adenosine dinucleotide; Naprt1: nicotinate phosphoribosyltransferase 1; Nam: nicotinamide; Nampt: nicotinamide phosphoribosyltransferase; Nadsyn1: NAD^+^ synthetase; NMN: nicotinamide mononucleotide; Nrk1: nicotinamide riboside kinase1; PACs: proanthocyanidins; Qprt: quinolinate phosphoribosyltransferase; Tdo2: tryptophan 2,3-dioxygenase; Trp: tryptophan.

NAD^+^ availability is determined by the relative rates of NAD^+^ biosynthesis and degradation. Although NAD^+^ is primarily synthesized by the *salvage* pathway in the liver^27^, our results indicate that PAC consumption increased the NAD^+^ levels by enhancing the *de novo* NAD^+^ biosynthesis pathway. Indeed, PAC increased the flux through the *de novo* biosynthesis pathway by overexpressing some of the key enzymes involved in this pathway and by increasing the precursor’s levels. In fact, the hepatic levels of Trp were robustly increased in a dose-response pattern, and it has been described that liver can increase the flux through the *de novo* pathway 40-fold when the levels of Trp or other NAD^+^ precursors increase^27^. Together, these results strongly suggest that PAC consumption fosters the use of Trp in the liver as the main precursor of NAD^+^, while maintaining the activity of the *salvage* biosynthetic pathway at basal levels (Fig. 8). Nampt expression was not altered by PACs at any dose. However, NMN levels, the product of the reaction catalyzed by Nampt, increased significantly at 50 mg of PACs, indicating that the actual Nampt activity in the liver of rats treated with PACs was sufficient to support the increased flux through the *salvage* pathway.

Interestingly, sirtuins use NAD^+^ as a co-substrate to remove acetyl moieties from lysines on histones and proteins, releasing Nam and *O*-acetyl-ADP-ribose. Generally, most sirtuins are activated when there is an energy deficiency and reduced carbohydrate energy sources, triggering cellular adaptations that improve metabolic efficiency. For example, SIRT1 activity increases during exercise^24^, caloric restriction or fasting^25^, all of which correlate with higher NAD^+^ levels, suggesting that NAD^+^ availability is the most important regulator of SIRT1 activity. In this study, PAC consumption increased SIRT1 activity in a dose-dependent pattern, according to the dose-response effect of PACs on the hepatic NAD^+^ levels. Notably, despite the levels of Nam, which is a potent inhibitor of Sirt1^28^, increasing dose-dependently following PACs consumption, SIRT1 activity was not inhibited because NAD^+^ levels increased higher than Nam levels, in the way that the ratio between NAD^+^/Nam remains in favor of NAD^+^ as the dose of PACs was raised.

In addition, PACs significantly reduced the expression of other non-SIRT NAD^+^ -consuming enzymes in the liver, such as PARP1 and CD38. In fact, diverse lines of research indicate that the pharmacological repression of PARP1 drives an increase in the NAD^+^ content and activation of SIRT1^29^,^30^, indicating that the competition between PARPs and SIRTs for NAD^+^ could be considered a metabolic determinant. Illustrating the opposing roles of both enzymes, PARP1 is required for the transcriptional co-activation of nuclear factor kappa B (NF-κB)^31^, while SIRT1 inhibits NF-κB activity through the deacetylation of RelA/p65^32^. Furthermore, SIRT1 also negatively regulates PARP1 transcription^33^. Thus, the results of this study indicate that PAC-mediated PARP-1 and CD38 repression could primarily contribute to SIRT1 activation.

Remarkably, PAC consumption also promoted *Sirt1* expression in a dose-response pattern. Several mechanisms control *Sirt1* expression, such as FOXO1, PPARα and PPARγ, among others. Together, these results indicate that the activation of SIRT1 by PACs was the result of increasing both the cellular NAD^+^ and SIRT1 concentrations. Therefore, PACs significantly foster SIRT1 activity. This property is not exclusive of PACs, and other polyphenols, such as resveratrol, can also potentially modulate SIRT1 activity both *in vitro* and *in vivo*^34^,^35^. However, the mechanism of SIRT1 activation by resveratrol has been debated, and recent studies show that resveratrol could not be a specific activator of SIRT1^36^,^37^.

In mammals, the SIRT family comprises seven proteins, SIRT1-7, each one with a specific cellular localization and function. Thus, we also evaluated whether PAC consumption could modulate the expression of *Sirt2* and *Sirt3*, which are primarily localized to the cytosol^38^ and the mitochondrial matrix^39^, respectively. However, the expression levels of *Sirt2* and *Sirt3* were not modified, indicating that PAC-mediated regulation of *Sirt1* expression is specific and not a general effect of PACs on the SIRT family.

SIRT1 is an important modulator of hepatic lipid metabolism by enhancing fatty acid oxidation, decreasing lipogenesis and modulating cholesterol levels through the activation of 5′ AMP-activated protein kinase (AMPK) and the deacetylation of both steroid response element binding protein 1 (SREBP1) and farnesoid X receptor (FXR)^40^. Accordingly, our group previously demonstrated that PACs reduced triglyceridemia in wild type mice but not in FXR-null mice, revealing that FXR is an essential mediator of the hypotriglyceridemic actions of these compounds *in vivo*^11^. In the liver, PACs down-regulated the expression of the transcription factor SREBP1 and several SREBP1-target genes involved in lipogenesis in an FXR-dependent manner. In addition, in cultured mammalian cells, PAC treatment increased the transactivation activity of FXR only when its natural agonist, the chenodeoxycholic acid, was present in the media^11^. Therefore, we can speculate that the direct transactivation of FXR by PACs could be mediated, at least in part, by the consecutive increase in NAD^+^ content and SIRT1 activation, although further studies are needed to confirm this hypothesis. In accord with this role, it has been widely demonstrated that hepatocyte-specific deletion of SIRT1 induces hepatic lipid accumulation by up-regulating the expression of lipogenic genes and reducing fatty acid oxidation^41^,^42^, whereas overexpression of SIRT1 protects against hepatic lipid accumulation and inflammation in response to moderate- and high-fat diets^43^. Interestingly, our results showed that PAC administration dose-dependently attenuated the hepatic lipid accumulation induced by an acute dietary fat challenge, due to the increased NAD^+^ levels and SIRT1 activity. Furthermore, we demonstrated that when the HepG2 cells were treated with an SIRT1 inhibitor, the triglyceride levels did not significantly decrease after the PAC treatment. Together, these results strongly suggest that PACs prevented the hepatic lipid accumulation through a SIRT1-mediated mechanism.

In conclusion, our current study provides the first *in vivo* evidence that the intracellular NAD^+^ levels, SIRT1 activity and lipid content in liver are globally modulated in a dose-dependent manner by chronic dietary supplementation with PACs. In view of these results, PACs activated *Sirt1* by both i) promoting NAD^+^ synthesis and ii) overexpressing *Sirt1*. These results suggest that PAC consumption could be a new valid strategy to enhance SIRT1 activity through the modulation of the *Parp1, Cd38* and *Sirt1* mRNA levels (major NAD^+^ -consuming enzymes) and the activation of the *de novo* NAD^+^ biosynthesis pathway. Interestingly, the PAC doses used in this experiment in rats are doses achievable in a normal human diet. Using a translation of animal to human doses^44^ and estimating the daily intake for a 70 kg human, the doses 5, 25 and 50 mg of PACs/kg of body weight correspond to an intake of 57, 284 and 560 mg of PACs/day, respectively. However, to translate these results from both rats and cells to humans, further studies are needed.

## Materials and Methods

### Grape seed PAC extract composition

The grape seed PAC extract was kindly provided by Les Dérives Résiniques et Terpéniques (Dax, France). According to the manufacturer, the extract is mainly composed of phenolic compounds (total content higher that 96%) with the phenolic content composed of monomers or flavan-3-ols (21.3%), dimers (17.4%), trimers (16.3%), tetramers (13.3%) and oligomers (5–13 units; 31.7%) of procyanidins. The phenolic composition of this extract was further analyzed by Quiñones, M. *et al*.^45^. Briefly, the analysis showed that the most important phenolic compounds contained in the extract were: catechin (58 μmol/g), epicatechin (52 μmol/g), epigallocatechin (5.50 μmol/g), epicatechin gallate (89 μmol/g), epigallocatechin gallate (1.40 μmol/g), dimeric procyanidins (250 μmol/g), trimeric procyanidins (1568 μmol/g), tetrameric procyanidins (8.8 μmol/g), pentameric procyanidins (0.73 μmol/g), and hexameric procyanidins (0.38 μmol/g).

### Animal handling

The investigation has been conducted in accordance with the ethical standards and according to the Declaration of Helsinki and has been approved by the Ethics Review Committee for Animal Experimentation of the Universitat Rovira i Virgili. Forty six-week-old male Wistar rats were purchased from Charles River Laboratories (Barcelona, Spain). The animals were singly housed in a 12 h light-dark-cycle at 22 °C and were provided a standard chow diet (Panlab 04, Barcelona, Spain) and tap water *ad libitum*. After one week of adaptation, the animals were randomly divided into four groups (n = 10) and supplemented with 0 (control group), 5, 25 or 50 mg of PACs/kg body weight (bw) for 21 days. The grape seed PAC extract was dissolved in 750 μL of commercial sweetened skim condensed milk (Nestle; 100 g: 8.9 g protein, 0.4 g fat, 60.5 g carbohydrates, 1175 kJ) at the appropriate concentrations. Before supplementation, all of the rats were trained to voluntarily lick the milk, and all groups were administered with the same volume of condensed milk for 21 days. The treatment was administered every day at 9:00 am. After 21 days of supplementation, the rats were fasted overnight. At 9:00 am, the rats were orally gavaged with lard oil (2.5 mL/kg bw) with the appropriate dose of the extract (0, 5, 25 or 50 mg/kg bw). Three hours later, the rats were sedated using a combination of ketamine (70 mg/kg bw, Parke-Davis, Grupo Pfizer, Madrid, Spain) and xylazine (5 mg/kg bw, Bayer, Barcelona, Spain). After anesthetization, the rats were exsanguinated from the abdominal aorta. The liver was excised and frozen immediately in liquid nitrogen and stored at −80 °C until the RNA and metabolites were extracted.

### ^1^H-NMR analysis

The liver metabolite extraction was performed according to the procedure described by Vinaixa M *et al*.^16^. Briefly, 50 mg of hepatic tissue was removed, flash-frozen, and mechanically homogenized in 1 mL of H_2_O/CH_3_CN (1/1). The homogenates were centrifuged at 5,000 × g for 15 min at 4 °C. The supernatants (hydrophilic metabolites) were separately lyophilized overnight and stored at −80 °C until further analysis. For the NMR measurements, the hydrophilic dried extracts were reconstituted in 600 μL of D_2_O containing 0.67 mM trimethylsilyl propionic acid (TSP). The samples were then vortexed, homogenized for 20 min, and centrifuged for 15 min at 6,000 × g at 4 °C. Finally, the redissolved samples were placed into 5 mm NMR tubes. The ^1^H-NMR spectra were recorded at 300 K on an Avance III 600 spectrometer (Bruker, Germany) operating at a proton frequency of 600.20 MHz using a 5 mm CPTCI triple resonance (^1^H, ^13^C, ^31^P) gradient cryoprobe. The one-dimensional ^1^H pulse experiments were performed using the nuclear Overhauser effect spectroscopy (NOESY) presaturation sequence (RD–90°–t1–90°–tm–90° ACQ) to suppress the residual water peak. Solvent presaturation with an irradiation power of 75 Hz was applied during the recycling delay (RD = 5 seconds) and mixing time (tm = 100 ms). The 90° pulse length was calibrated for each sample and varied from 6.57 to 6.99 ms. The spectral width was 12 kHz (20 ppm), and a total of 256 transients were collected into 64 k data points for each ^1^H spectrum. The exponential line broadening applied before Fourier transformation was 0.3 Hz. The frequency domain spectra were phased and baseline-corrected using TopSpin software (version 2.1, Bruker).

### LC-QqQ-MS/MS analysis

NAD^+^ and its related metabolites and end-products, Nam, NMN, Na, Nr, NaMN, NaAD, nicotinamide *n*-oxide, nicotinuric acid, *n*-methylnicotinamide, and Trp, were measured in the liver samples using an LC-QqQ-MS/MS system consisting of an Agilent HPLC 1200 Series (Agilent Technologies, Palo Alto, USA). Briefly, 60 mg of the lyophilized liver samples were vigorously vortexed in 0.5 mL of physiological saline for 30 seconds, followed by 30 seconds of ultrasonication in a Vibra Cell (Sonics, Newton, USA). Then, 0.5 mL of acetone was added and the samples were centrifuged at 10,000 × g for 15 min at 20 °C. This procedure was repeated twice; the upper phases were mixed and evaporated under nitrogen flow to dryness. Finally, the dried samples were dissolved in 100 μL of the mobile phase at the initial conditions and injected into the liquid chromatograph (LC). A Scherzo SM-C18 (3 μm; 150 mm × 2 mm i.d.; Imtakt, Japan) was used to perform the analysis. The LC was coupled to a triple quadrupole (QqQ) mass spectrometer (MS) 6410 (Agilent Technologies, Palo Alto, USA).

### Cell culture and treatments

The HepG2 human hepatoma cells obtained from the American Type Culture Collection (ECACC code 85011430) were cultured in DMEM containing 25 mM 4-(2-hydroxyethyl)-1-piperazineethanesulfonic acid (Hepes) buffer, 10% (vol/vol) foetal bovine serum (FBS), 2 mM l-glutamine, 0.1 mM non-essential amino acids (NEAA), 100 U/mL penicillin, and 100 mg/mL streptomycin, 2.5 mg/L Amphotericin B solution at 37 °C in a humidified atmosphere containing 5% CO_2_, with medium changes three times a week. The cells were incubated in 12-well plates at a density of 5 × 10^6^ cells/well for 48 hours or until the cells were 70% confluent before starting the experimental treatments. To establish a hepatocellular model of fat-overloading, the HepG2 cells were incubated with a mixture of 0.5 mM palmitate (P9767, Sigma-Aldrich, Madrid, Spain) combined with 0.5% fatty acid free-BSA (A3803, Sigma-Aldrich) and 30 mM glucose (131341.1211, Panreac, Barcelona, Spain) for 48 hours. For the last 24 hours of incubation, the cells were treated with 100 mg/L PACs in the presence or absence of 10 or 100 μM sirtinol (10523, Cayman Chemical, Tallin, Estonia), a SIRT1 inhibitor. The treatments were carried out in triplicate in three independent experiments. Finally, the cell lysates were collected and stored at −80 °C for the triglyceride and RT-qPCR analyses.

Cytotoxicity of grape seed PACs on HepG2 cell line was evaluated previously and doses below 350 mg/L were safe^46^. Therefore, 100 mg PACs /L allows to do *in vitro* experiments with high levels of PACs without affecting HepG2 cell viability. Additionally, we have used this dose of PACs in many other studies, showing that this dose is effective at modulating gene expression and metabolism in HepG2^47^,^48^,^49^. Based on the composition of the extract, the mean molecular weight of grape seed PACs extract used in this experiment is 780 g/mol; thus, 100 mg PACs /L is equivalent to 0.13 μM.

### qRT-PCR analysis

The total RNA was extracted from the liver and cultured cells using TRIzol reagent and an RNeasy Mini Kit (Qiagen, Valencia, CA) according to the manufacturer’s protocols. The quantity and purity of RNA was measured using a NanoDrop 1000 Spectrophotometer (Thermo Scientific). Samples with the RNA concentration (A260/A280 ≥ 1.8 ng/μl) and purity (A230/A260 ≥ 2.0 ng/μl) were selected. Additionally, RNA integrity was assessed by denaturing gel electrophoresis stained with SYBR Green dye (BioRad). Complementary DNAs were generated using the High-Capacity complementary DNA Reverse Transcription Kit from Life Technologies (Uppsala, Sweden). The relative mRNA expression levels of the selected genes were assessed in the liver and normalized to the cyclophilin peptidylprolyl isomerase A (*Ppia*) mRNA levels. The forward (F) and reverse (R) primers used in this study can be found as Supplementary Table S1 online. Briefly, a total of 10 ng of the cDNAs was subjected to quantitative RT-PCR amplification using the SYBR Green PCR Master Mix from Bio-Rad (Barcelona, Spain). The reactions were run on a CFX96 real-time system-C1000 Touch Thermal Cycler (Bio-Rad); the thermal profile settings were 50 °C for 2 min, 95 °C for 2 min, and then 40 cycles at 95 °C for 15 s and 60 °C for 2 min. Finally, the fold change in the mRNA levels was calculated and normalized to the linear form by the 2^−∆∆Ct^ calculation^50^. For that, only samples with a quantification cycle lower than 30 were used for fold change calculation.

### SIRT1 activity assay

Hepatic SIRT1 activity was determined according to the method described by Becatti, M. *et al*.^51^ with some modifications. Liver extracts were obtained using a mild lysis buffer (50 mM Tris-HCl pH 8, 125 mM NaCl, 1 mM DTT, 5 mM MgCl2, 1 mM EDTA, 10% glycerol, and 0.1% NP40). SIRT1 activity was measured using a SIRT1 direct fluorescent screening assay kit (Cayman, Ann Arbor, MI), following the manufacturer’s protocol. Briefly, a total of 25 μl of assay buffer (50 mM Tris–HCl, pH 8.0, containing 137 mM NaCl, 2.7 mM Kcl, and 1 mM MgCl2), 5 μl of liver extract, and 15 μl of substrate (Arg-His-Lys-Lys(ɛ-acetyl)-)-7-amino-4-methylcoumarin) solution were added to all wells. The fluorescence intensity was monitored every 2 min for 1 h using the fluorescence plate reader Bio-Tek FLx800, applying an excitation wavelength of 355 nm and an emission wavelength of 460 nm. The results are expressed as the rate of reaction for the first 30 min, when there was a linear correlation between the fluorescence and this period of time.

### Lipid analysis

The liver lipids (0.5 g) were extracted using the Folch method^52^. An aliquot of the extract was subjected to gravimetric analysis to measure the total lipid concentrations. The remaining extract was allowed to evaporate under a nitrogen flow and dissolved in 1 mL of lipoprotein lipase buffer, containing 1,4-Piperazinediethanesulfonic acid disodium salt ((P3768, Sigma-Aldrich), MgCl_2_·6 H_2_O (M9272, Sigma-Aldrich), albumin-free fatty acids (A8806, Sigma-Aldrich) and 0.1% sodium dodecyl sulfate (L3771, Sigma-Aldrich). The triglycerides and cholesterol concentrations in the dissolved extract and in the cultured cell lysates were measured using QCA enzymatic colorimetric kits (QCA, Barcelona, Spain) according to the manufacturer’s protocols.

### Data analysis and statistical methods

The pure compound references in BBioref AMIX (Bruker), HMDB and Chenomx databases were used for the ^1^H NMR metabolite identification. In addition, we assigned the metabolites by ^1^H–^1^H homonuclear correlation (COSY and TOCSY) and ^1^H–^13^C heteronuclear (HSQC) 2D NMR experiments and by correlation with the pure compounds run in-house. After baseline correction, the specific ^1^H NMR regions identified in the spectra were integrated using the AMIX 3.9 software package (Bruker, GmBH) and the resulting dataset was explored through principal component analysis (PCA). LC-QqQ-MS/MS data acquisition was performed using Masshunter software. The analyses were performed in positive electrospray ionization (ESI+) mode. The selected reaction monitoring (SRM) transitions and the individual fragmentor voltage and collision energy for each compound were evaluated using commercial standards to obtain the best instrumental conditions. Two transitions were acquired for each compound: one for quantification and a second for confirmation purposes (data not shown). The quality parameters of the analytical method (linearity, recovery, accuracy, reproducibility, limit of detection and limit of quantification) were evaluated to confirm the reliability of the method. These parameters were determined by spiking the basal liver extract with known concentrations of the standards. All of the reported values are expressed as the means ± SE (standard error) and were analyzed using the IBM SPSS for Windows statistical package (v.21.0). The differences between groups were initially calculated using ANOVA (single-factor or two factors) and the Mann–Whitney *U*-test; Student’s unpaired *t* test and Fisher’s exact test were used as necessary. Spearman’s correlation coefficients were also used to evaluate the degree of association between variables. Finally, a multiple linear regression model was fitted to evaluate which gene expression and metabolomic variables were independently associated with the hepatic fat content. The P-values for the gene expression analyses were calculated using the 2^−∆∆Ct^ method. *P*-values < 0.05 were considered statistically significant.

## Additional Information

**How to cite this article**: Aragonès, G. *et al*. Dietary proanthocyanidins boost hepatic NAD^+^ metabolism and SIRT1 expression and activity in a dose-dependent manner in healthy rats. *Sci. Rep.* **6**, 24977; doi: 10.1038/srep24977 (2016).

## Supplementary Material

Supplementary Information
