# Supplementary material for: Dietary proanthocyanidins boost hepatic NAD+ metabolism and SIRT1 expression and activity in a dose-dependent manner in healthy rats
Source: Sci Rep. 2016 Apr 22;6:24977. doi: 10.1038/srep24977 (PMC4840337; doi:10.1038/srep24977)
Supplement: Supplementary Information [file srep24977-s1.doc]

**Dietary proanthocyanidins boost hepatic NAD^+^ metabolism and SIRT1 expression and activity in a dose-dependent manner in healthy rats**

Gerard Aragonès, Manuel Suárez, Andrea Ardid-Ruiz, Maria Vinaixa, Miguel A Rodríguez, Xavier Correig, Lluís Arola & Cinta Bladé

| Supplementary Table 1. A summary of the rat-specific primer sequences used for qRT-PCR analysis. Ten nanograms of the cDNAs were subjected to quantitative amplification using the SYBR Green PCR Master Mix from Bio-Rad (Barcelona, Spain). The reactions were run on a CFX96 real-time system-C1000 Touch Thermal Cycler (Bio-Rad). The thermal profile settings were 50°C for 2 min, 95ºC for 2 min, and then 40 cycles at 95°C for 15 s and 60°C for 2 min | | | | | |
| --- | --- | --- | --- | --- | --- |
| Rat genes | Alignment  sequence | Direction | Primer sequences (5’-3’) | Primer length (nucleotides) | Tm (ºC) |
| *Cd38* | NM_013127.1 | Fw | CAG CAC CTT TGG AAG TGT GG | 20 | 53 |
|  |  | Rv | CAG GTC GGT AGT TAT CCT GGC | 21 | 53 |
|  |  |  |  |  |  |
| *Gadd45* | NM_024127.2 | Fw | TAC ATG GAT CAG TGG GTG CC | 20 | 53 |
|  |  | Rv | TGG GGA GTG ACT GCT TGA GT | 20 | 52 |
|  |  |  |  |  |  |
| *Nadsyn1* | NM_181480.1 | Fw | GCC CTT TCG GTC AGT GAA GA | 20 | 54 |
|  |  | Rv | GTC GAA GGA AGT CCC AGA GC | 20 | 53 |
|  |  |  |  |  |  |
| *Nampt* | NM_177928.3 | Fw | CTC TTC ACA AGA GAC TGC CG | 20 | 53 |
|  |  | Rv | TTC ATG GTC TTT CCC CCA CG | 20 | 52 |
|  |  |  |  | 2 |  |
| *Naprt1* | NM_207609.1 | Fw | TAG CCC AAA AGG GCA GTG AG | 20 | 54 |
|  |  | Rv | CAG CTT GTA GAC ACA GCC CA | 20 | 51 |
|  |  |  |  |  |  |
| *Nrk1* | NM_001024292.1 | Fw | CCT GGA CGG AAC AAG GTC TG | 20 | 54 |
|  |  | Rv | CCT TCA AGG GTC CGA ATC CA | 20 | 56 |
|  |  |  |  |  |  |
| *Parp1* | NM_013063.2 | Fw | ACC GAG TGG AGT ACG CTA AG | 20 | 49 |
|  |  | Rv | GAA CAT GGG TGA CTG CAC CA | 20 | 54 |
|  |  |  |  |  |  |
| *Qprt* | NM_001009646.1 | Fw | CAC CAT GGA CCC TGA AGG TCT G | 22 | 58 |
|  |  | Rv | TAG CCC GGT AAC GGA CGC AA | 20 | 59 |
|  |  |  |  |  |  |
| *Sirt1* | XM_006256146.2 | Fw | TTG GCA CCG ATC CTC GAA | 18 | 54 |
|  |  | Rv | ACA GAA ACC CCA GCT CCA | 18 | 52 |
|  | | | | | |
| *Sirt2* | NM_001008368.1 | Fw | AAA CCT CCC ACC TTC ACT GC | 20 | 53 |
|  |  | Rv | GGA TAA GGT AGC GAG TGC GA | 20 | 52 |
|  |  |  |  |  |  |
| *Sirt3* | NM_001106313.2 | Fw | TGT GGG GTC CGG GAG TAT TA | 20 | 54 |
|  |  | Rv | CCA CCA TGA CCA CAA CCC TA | 20 | 53 |
|  |  |  |  |  |  |
| *Sod2* | NM_017051.2 | Fw | CAC CGA GGA GAA GTA CCA CG | 20 | 52 |
|  |  | Rv | TGG GTT CTC CAC CAC CCT TA | 20 | 54 |
|  |  |  |  |  |  |
| *Tdo2* | NM_022403.2 | Fw | CTC CTG GGA CGC ATC ACT AC | 20 | 52 |
|  |  | Rv | AAG TCC TCC TTT GCT GGC TC | 20 | 53 |
|  |  |  |  |  |  |
| *Ucp2* | NM_019354.3 | Fw | GAG AGT CAA GGG CTA GCG C | 19 | 54 |
|  |  | Rv | GCT TCG ACA GTG CTC TGG TA | 20 | 51 |
| **Abbreviations:** *Cd38*, CD38 molecule; *Gadd45*, growth arrest and DNA-damage-inducible; *Nadsyn1*, NAD synthetase 1; *Nampt*, nicotinamide phosphoribosyltransferase; *Naprt1*, nicotinate phosphoribosyltransferase; *Nrk1*, nicotinamide riboside kinase 1; *Parp1*, poly (ADP-ribose) polymerase 1; *Qprt*, quinolinate phosphoribosyltransferase; *Sirt1*, sirtuin 1; *Sirt2*, sirtuin 2; *Sirt3*, sirtuin 3; *Sod2*, superoxide dismutase 2; *Tdo2*, tryptophan 2,3-dioxygenase; *Ucp2*, uncoupling protein 2. | | | | | |
